# Reduced Invasiveness of Common Ragweed (*Ambrosia artemisiifolia*) Using Low-Dose Herbicide Treatments for High-Efficiency and Eco-Friendly Control

**DOI:** 10.3389/fpls.2022.861806

**Published:** 2022-05-12

**Authors:** Hanyue Wang, Tong Liu, Wenxuan Zhao, Xuelian Liu, Mingming Sun, Pei Su, Jun Wen

**Affiliations:** ^1^Xinjiang Production and Construction Corps Key Laboratory of Oasis Town and Mountain-Basin System Ecology, College of Life Sciences, Shihezi University, Shihezi, China; ^2^Office of Locust and Rodent Control Headquarters of Ili Kazak Autonomous Prefecture, Yining, China

**Keywords:** chemical control, fitness, interspecific competition, species diversity, tradeoff

## Abstract

Common ragweed (*Ambrosia artemisiifolia*) is an invasive annual weed that invades heavily disturbed habitats and natural habitats less disturbed by human activities with native plant species in need of protection. Achieving effective control of *A. artemisiifolia* for the protection of native organisms and the local ecological environment is an ongoing challenge. Based on the growth and development characteristics of *A. artemisiifolia*, we examined the effectiveness of herbicides in controlling this species and the optimal time for application in the field with the aim of reducing herbicide dosage. Additionally, we analyzed whether the efficiency of low-dose applications for controlling this species might improve with increasing native plant species richness. Our findings indicate that aminopyralid (33 g ai ha^−1^) was the most suitable herbicide for chemical control of *A. artemisiifolia,* with optimum application time being during vegetative growth (BBCH 32–35). Application of aminopyralid was found to kill approximately 52% of *A. artemisiifolia* plants, and more than 75% of the surviving plants did not bloom, thereby reducing seed yield of the population by more than 90%. Compared with the application of high-dose herbicide, the phytotoxicity of aminopyralid to native plants at the applied dose was substantially reduced. After 2 years of application, the relative coverage of *A. artemisiifolia* significantly decreased, with few plants remaining, whereas the relative coverage of native plants more than doubled, representing an eco-friendly control. Further, there was an increase in the *A. artemisiifolia* control rate in the plant community with higher native plant species richness at the same herbicide rates and a reduction in seed yield of *A. artemisiifolia*. Our findings help toward developing control measures to reduce the invasiveness of *A. artemisiifolia* with low-dose herbicides meanwhile protecting native plants, and then using the species richness of native plant communities to indirectly promote the effectiveness of low-dose herbicide application.

## Introduction

Common ragweed (*Ambrosia artemisiifolia* L.) is an invasive annual weed native to North America that has become widely distributed worldwide (Montagnani et al., 2017). *A. artemisiifolia* can produce large amounts of allergenic pollen becoming a threat to public health (Schaffner et al., 2020). Furthermore, *A. artemisiifolia* can invade farmland and greatly reduce crop yields (Pinke et al., 2019; Hall et al., 2021). Overall, the negative impacts of this species on agriculture and human health have been estimated to cause economic losses of up to 4.5 billion Euros per year in Europe (Bullock et al., 2012). Therefore, there is an urgent need to reduce the spread of *A. artemisiifolia* to minimize these negative effects.

Effective control of *A. artemisiifolia* has long been a challenge, particularly in the context of ensuring eco-friendliness and low cost. Herbicide resistance poses another difficult challenge, especially for European and North American countries (Cseh et al., 2009; Barnes et al., 2017; Phillips, 2020). Chemical control is often used to limit *A. artemisiifolia* invasion in farmlands because it can effectively kill plants rapidly or reduce seed production (Bae et al., 2017). However, excessive use of herbicides, such as glyphosate, has resulted in the development of herbicide-resistant populations (Powles, 2008). Further, physical control methods are time-consuming, labor-intensive, and relatively ineffective (Katz et al., 2014; Lommen et al., 2018). In recent years, biological control has achieved effective control, but host-specificity tests still need to be researched to limit the accidental introduction of invasive biocontrol species. Besides, other agents might be needed to cover cooler regions (Mouttet et al., 2018; Schaffner et al., 2020; Sun et al., 2022). In China, for example, although *Ophraella communa* LeSage can effectively control *A. artemisiifolia* in southern China, this insect rarely survives the winter in northern China (Zhou et al., 2010, 2015).

Thus, for effective and rapid control of species through simple techniques and at a low cost, chemical control must be reconsidered. *A. artemisiifolia* can be invasive in areas heavily disturbed by human activities, including roadside verges, wastelands, railway embankments, construction sites, quarries, at the edge of croplands and arable fields (Bassett and Crompton, 1975; Milakovic et al., 2014; Essl et al., 2015; Montagnani et al., 2017; Lemke et al., 2019). This species can also be found in natural habitats with less human activity (Fumanal et al., 2008; Dong et al., 2020). Therefore, low doses of herbicide are needed to reduce *A. artemisiifolia* fitness while minimizing harm to native plants, and to further inhibit the fitness of *A. artemisiifolia* through competitive exclusion by native plant species (Fargione and Tilman, 2005; Oakley and Knox, 2013).

*A. artemisiifolia* was first reported in the Yili River Valley (Xinjiang, China) in 2010, and by 2017, its distribution area in this newly invaded region had reached approximately 101,500 ha (Dong et al., 2017, 2020). Thus, highly effective control methods are needed to prevent this species from spreading further. However, control strategies that rely on the use of chemical herbicides at high doses can lead to environmental problems, such as harming native plant species and long-term pollution (Kujawa et al., 2017).

Effective control is not necessarily achieved by killing the invading plant. In the case of *A. artemisiifolia*, a single plant can produce between 3,000 and 60,000 seeds, depending on plant size (Dickerson and Sweet, 1971; Milakovic and Karrer, 2016). Although *A. artemisiifolia* is distributed worldwide, its interspecific competitiveness is not always stronger than that of other species (Leskovšek et al., 2012). Indeed, *A. artemisiifolia* seems to readily colonize an area through characteristics related to seed production (propagule pressure), germination, and seedling survival (Kempel et al., 2013). Studies have shown that *A. artemisiifolia* undergoes a rapid growth phase from the vegetative stage to the reproductive stage (Zhao et al., 2021), when biomass accumulation is higher in short time, and that herbicide application at this time may activate its growth-defense trade-off mechanism (Coley et al., 1985; Figueroa-Macías et al., 2021), i.e., the activation of defense mechanisms at the cost of suspending growth. This reduces the effective accumulation of biomass in each organ and ultimately reduces seed production (Zhao et al., 2021), or might even render the plant unable to produce any seeds at all. Moreover, this process is not aimed at killing all *A. artemisiifolia* plants, so the required herbicide dosage should be lower than the guideline dosage. The treatment may simply result in hindering normal growth and development, accompanied by a stronger interspecific competitive effect from other plant species. Studies on *A. artemisiifolia* seed banks have shown that without new seed replenishment for two consecutive years, the existing seed bank will be depleted by more than 75% (Dong et al., 2021). Therefore, effective control may be achieved in a short period without the need to repeatedly apply low-dose herbicides over a long period, which may allow *A. artemisiifolia* to develop resistance to the selected herbicide.

Our study aimed to determine whether *A. artemisiifolia* populations can be effectively controlled by reducing seed production with low-dose herbicide application, rather than by killing all plants. Several experiments were conducted in the Yili Valley of Xinjiang, China, which is heavily invaded with *A. artemisiifolia*, to determine: (1) the most effective herbicide, (2) the most effective time for herbicide application, (3) the relationship between *A. artemisiifolia* invasiveness and native species richness, and (4) the effect on restoration of native plants after multiple years of control.

## Materials and Methods

### Study Sites

The study area, located in the Yili River Valley (42°14′16′′–44°53′30′′N, 80°09′42″–84°56′50″E), is west of Tianshan Mountain in Xinjiang, China, which is the wettest area in Xinjiang. The average annual temperature in the area is 10.4°C and the total annual precipitation is 417.6 mm (Dong et al., 2020).

**Figure 1:**
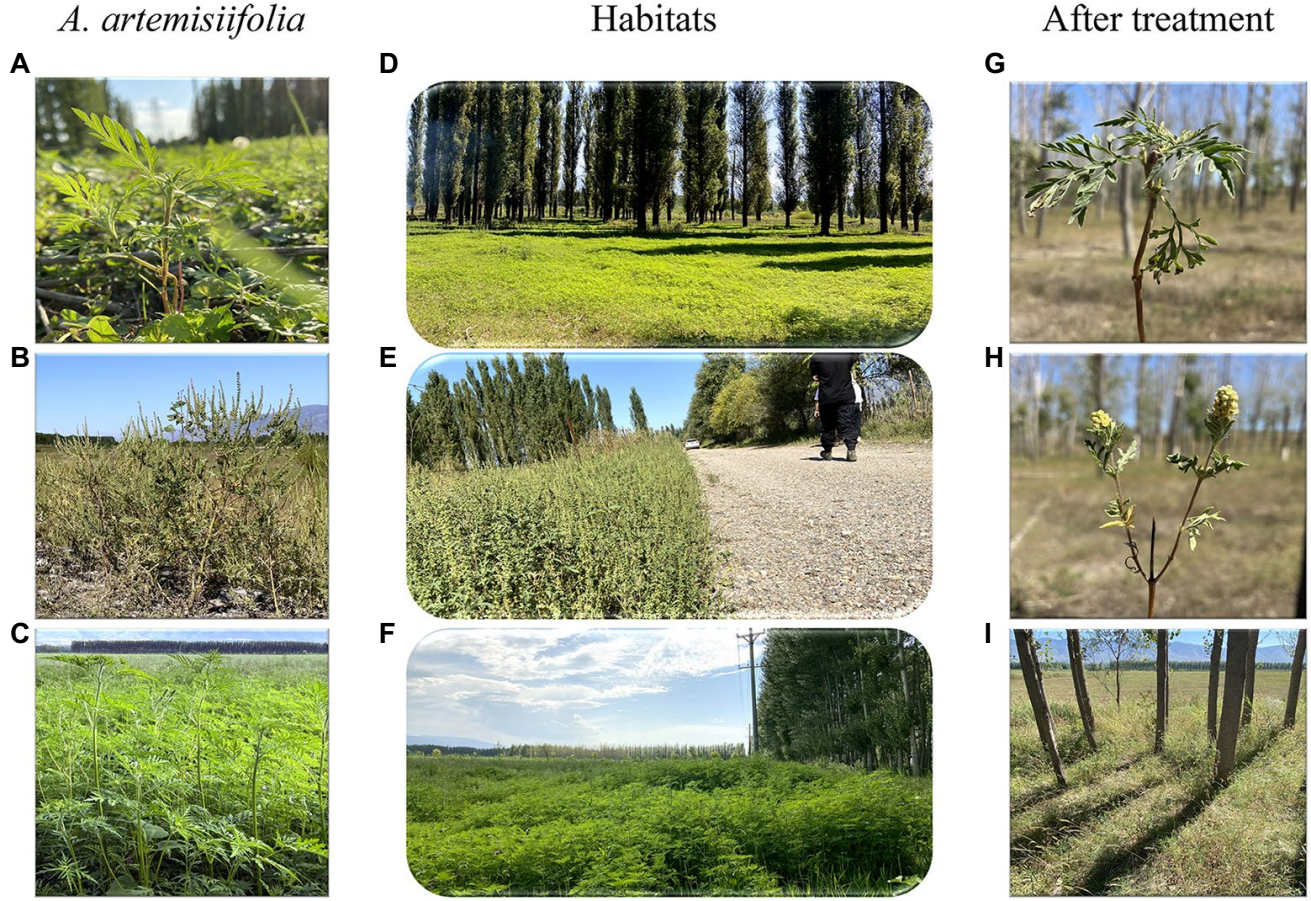
Study site and herbicide effects on habitat and plants. Panels **(A–C)** were *Ambrosia artemisiifolia* in woodland **(D)**, roadside **(E)**, and farmland **(F)** habitats, respectively. Panel **(G)** indicated that *A. artemisiifolia* couldnot flower after herbicide application. Panel **(H)** indicated that although *A. artemisiifolia* could flower after herbicide application, it produced only few seeds. Panel **(I)** showed that *A. artemisiifolia* disappeared from the habitat after 2 years of herbicide application.

Three habitats commonly invaded by *A. artemisiifolia*, namely woodland (43°28′49″N, 83°20′16″E), roadside (43°27′48” N, 83°28′29″ E), and farmland edge (43°32′17″N, 83°15′37″E, hereafter referred to as farmland), were selected for the study (Figure 1), harboring 20, 22, and 18 plant species, respectively (see Supplementary Table 1 for details of these species). Perennial herbaceous plants comprised 55, 41 and 50% of all plant species in woodland, roadside and farmland habitats, respectively, with the remainder consisting of annuals and biennials. There were differences in species richness and community coverage across the experimental plots, where the number of *A. artemisiifolia* plants at different growth stages decreased abruptly from the seedling to the vegetative growth stage (Table 1). The farmland habitat was irrigated multiple times during the growing season, and there was a crop rotation of wheat and corn throughout the year. The roadside habitat was defined as an approximately 6-m-wide space between the main road and an approximately 5-m-wide windbreak belt separating the roadside space from the adjacent farmlands. The woodland was along the Kunes River, where trees were planted approximately 30 years ago to form an embankment, after which, natural succession has occurred unchecked up until the time when this study was conducted.

**Table 1:** Characteristics of the three studied habitats.

Habitats	Total number of species	Species in block	Coverage	Population density of *Ambrosia artemisiifolia* (plants m^−2^)		
				BBCH 14	BBCH 32–35	BBCH 60–61
Woodland	20	7.59 ± 0.74	0.73 ± 0.06	2,984 ± 281	819 ± 129	443 ± 93
Roadside	22	6.14 ± 0.39	0.48 ± 0.04	1,428 ± 355	398 ± 156	218 ± 47
Farmland	18	5.2 ± 0.82	0.76 ± 0.01	1986 ± 351	117 ± 18	72 ± 12

Values are means ± SE. BBCH classification criteria refer to Hess et al. (1997). BBCH 14, BBCH 32–35, and BBCH 60–61 are in the seedling stage, vegetative growth stage and reproductive growth stage of *Ambrosia artemisiifolia*.

### Herbicide Screening

Herbicide screening was conducted in woodland habitats invaded by *A. artemisiifolia* a long time ago, with few native plants remaining. Four randomized blocks were established at random, comprising 12 (5 × 6 m) plots each, for a total of 11 herbicide treatments and a control. Three fixed quadrats (1 × 1 m) were set up in each plot for index observation and data collection.

The 11 herbicides used in the experiment are listed in Table 2. Two non-selective herbicides, glyphosate and glufosinate, were included in the herbicide screening test to verify the control effectivity of non-selective herbicides on *A. artemisiifolia*, and whether *A. artemisiifolia* plants at the study site show resistance to glyphosate.

**Table 2:** The 11 herbicides used in the experiment.

Herbicides	Application rates (g ai ha^−1^)	HRAC group	Site/mode of action
2,4-D	428	O	Auxin mimics
MCPA-Na	1008	O	Auxin mimics
Fluroxypyr	180	O	Auxin mimics
Clopyralid	113	O	Auxin mimics
Aminopyralid	110	O	Auxin mimics
Penoxsulam	22.5	B	Inhibition of ALS
Bentazone	1080	D	PSl electron diversion
Oxyfluorfen	360	E	Inhibition of PPO
Glyphosate	900	G	Inhibition of EPSP synthase
Glufosinate	900	H	Inhibition of glutamine synthetase
Dicamba	216	P	Auxin transport inhibitors

HRAC group and Site/mode of action classification basis from “Herbicide resistance action committee: herbicide classification” (Beffa et al., 2019).

A herbicide screening test was conducted in late July 2018 and applied upon the appearance of male flower buds (BBCH 51, Bae et al., 2017). Eleven commercial herbicides were prepared according to manufacturer instructions to select the most effective herbicide. A hand-powered knapsack sprayer with a capacity of 16 l was used for herbicide application, with a nozzle size of 1.0 mm, a working pressure of approximately 0.35 MPa, and a flow rate of approximately 400–540 ml min^−1^. Spraying was performed uniformly, with a spray volume of 450 l ha^−1^, and a spray volume of approximately 1.35 l per plot. After 1 month, plant control rate was evaluated for each treatment group, and all *A. artemisiifolia* plants in the three fixed quadrats in each plot were clipped at the seed stage to calculate seed yield.

### Determination of the Optimum Time and Dose of Herbicide Application

The effects of herbicide dose and time of application on the control of *A. artemisiifolia* were evaluated. Nine blocks were randomly set up in each of the three habitats (woodland, roadside, and farmland). Three blocks were selected at a time for herbicide spraying of *A. artemisiifolia* during each growth stage (seedling, vegetative growth, and reproductive growth). Each block comprised six (5 × 6 m) plots for a total of five dose treatments and a control treatment. Three fixed quadrats (1 × 1 m) were set up in each plot for index observation and data collection.

Following the herbicide screening test in 2018, aminopyralid was identified as the most effective herbicide (Table 3). To determine the best time and dose for application, aminopyralid tests were performed in 2019, for which, three blocks were randomly selected at the seedling (BBCH 14), vegetative growth (BBCH 32–35, in mid-June, when rapid growth of *A. artemisiifolia* was recorded, with an average plant height of approximately 20, 35, and 70 cm in woodland, roadside, and farmland, respectively), and the reproductive growth stages (BBCH 60–61, in late July). Aminopyralid dose treatments included X (110 g ai ha^−1^), 0.66X (73 g ai ha^−1^), 0.45X (49 g ai ha^−1^), 0.30X (33 g ai ha^−1^), and 0.20X (22 g ai ha^−1^). The GR_50_ (herbicide application rate required for 50% growth reduction) of aminopyralid for *A. artemisiifolia* was 33 g ai ha^−1^, as per results from the previous experimental study and the current study (Supplementary Table 2). The time of each application was evaluated using three blocks in each habitat. Except for measurement of aboveground biomass, all other data, including plant density, mortality rate, seedling regeneration, and plant height of *A. artemisiifolia* were collected from three fixed quadrats in each plot. *A. artemisiifolia* plant density was measured before each application. At 30 d after each treatment, the mortality rate, seedling regeneration, plant height, and aboveground biomass of *A. artemisiifolia* were recorded, along with the aboveground biomass of the native species. After herbicide treatment during the vegetative growth stage, the number of *A. artemisiifolia* plants that did not bloom was counted (n = 30). All the *A. artemisiifolia* plants in the three fixed quadrats in each plot were harvested during the seed stage to calculate seed yield.

**Table 3:** Effects of different herbicides on control rate and seed yield of *Ambrosia artemisiifolia*.

Herbicides	Application rates (g ai ha^−1^)	Control rate	Reduce seed yield
2,4-D	428	35% ± 5%^d,e^	75% ± 5%^b^
MCPA-Na	1008	10% ± 2%^g^	45% ± 3%^e,f^
Glufosinate	900	56% ± 5%^c^	94% ± 1%^a^
Glyphosate	900	58% ± 4%^c^	97% ± 1%^a^
Fluroxypyr	180	71% ± 8%^b^	63% ± 8%^d^
Bentazone	1080	17% ± 6%^g^	42% ± 7%^f^
Oxyfluorfen	360	25% ± 5%^f^	72% ± 3%^b,c^
Penoxsulam	22.5	41% ± 4%^d^	52% ± 5%^e^
Clopyralid	113	73% ± 7%^b^	67% ± 3%^c,d^
Dicamba	216	33% ± 3%^e^	65% ± 9%^c,d^
Aminopyralid	110	90% ± 6%^a^	98% ± 1%^a^

Values are means ± SE. Different letters indicate significant differences (*p* < 0.05) using the least significant difference test.

### Effects of Native Plant Species Richness on Herbicide Efficacy

After the optimal application period was determined, in-depth analysis of the data was undertaken to determine the relationship between native species richness and *A. artemisiifolia* fitness, and whether effective control might be achieved with lower doses. During the vegetative growth stage of *A. artemisiifolia*, when other plants in the community were growing and could be identified, the species richness of each habitat (number of species in habitat), the species richness (number of species in each plot) and the number of individuals in each plot were calculated.

A linear mixed model analysis was used to examine whether under the same aminopyralid dose, there was an increase in the species richness of native plants, and an improved control effect had been achieved.

### Restoration of Native Plants

After the optimal application time and dose had been determined, the experiment continued in 2020 using the appropriate application time and dose. Aminopyralid treatments were applied in 2019 and 2020. Changes in the relative cover of *A. artemisiifolia*, the native plants and the bare patch were examined in each group of blocks annually before chemicals were applied. Data collection was also undertaken in 2021 to assess the effect of the herbicide on the control of *A. artemisiifolia* and on the restoration of native plants.

### Statistical Analysis

The plant community was divided into two parts: *A. artemisiifolia* and native plants. Because of the random distribution of native plant species, it is difficult to quantitatively observe the response of each plant species before and after herbicide treatment. The aboveground biomass was, therefore, used to represent the impact of herbicides on native plants because some plants were adversely affected but not killed by the herbicide treatment. The interannual variability of native plants under herbicide influence was expressed as relative coverage at the community level (represented by blocks in the paper). To identify the most effective herbicide and the most suitable control period, one-way ANOVA and Fisher’s least significant difference (LSD) multiple comparisons of means were used to analyze the effects of the 11 herbicides on *A. artemisiifolia* plants control and seed yield (Equation 1), compared to the unsprayed control. Similarly, the effects of herbicide treatment and the application period on *A. artemisiifolia* growth, mortality rate (plant height, aboveground biomass), reproduction (number of flowering plants and seed yield), and native plant species growth (aboveground biomass), were calculated.


(1) $$E=\frac{CK_{1}-Pt_{1}}{CK_{1}}\times 100\%$$

where *E* stands control rate of *A. artemisiifolia* plants or seed yield reduction rate.

$CK_{1}$ stands the number of *A. artemisiifolia* plants (or seeds) in the control group without herbicide application. $Pt_{1}$ stands for the number of *A. artemisiifolia* plants (or seeds) in the herbicide application treatment.

To determine the effects of native species richness and number of individuals on *A. artemisiifolia* fitness, linear mixed model analysis was conducted with the lmerTest package in R 3.6.3 using data on optimal application time. *A. artemisiifolia* plant control and seed yield were used as dependent variables, while habitat and herbicide dose were the fixed effects, and species richness and the number of individuals in each plot and block were defined as random effects. To examine the impact of species richness on *A. artemisiifolia* seed yield, correlation analyses of “seed yield” and “species richness” data were conducted for each of the three habitats. The reasons are as follows: (1) Under the same chemical dose treatment, seed yield was found to be different with the assumption of a negative correlation. (2) Seed yield of *A. artemisiifolia* varied significantly among habitats; thus, the same plant species richness may correspond with different seed numbers. Therefore, some factors showed a significant correlation, while others showed no significant correlation (Table 4).

**Table 4:** Linear mixed models were used to analyze the influence of each factor on *A. artemisiifolia* fitness.

Model	Predictor	Effects		Sum Sq	NumDF	*F*-value	*P*
M1	Control rate of each treatment	Fixed effects	Habitat	0.01323	2	0.96	0.3916
			Dose	0.3684	1	53.459	< 0.001
		Random effects	Block	/	/	/	0.0846
			Species richness	/	/	/	< 0.001
			Individual number	/	/	/	0.9999
M2	Seed yield of each treatment	Fixed effects	Habitat	4,280,099	2	33.561	< 0.001
			Dose	1,200,256	1	19.520	< 0.001
		Random effects	Block	/	/	/	0.4319
			Species richness	/	/	/	0.9999
			Individual number	/	/	/	0.6127

*A. artemisiifolia* plant control and seed yield were used as dependent variables, while habitat and aminopyralid dose were the fixed effects, and species richness and the number of individuals in each plot and block were defined as random effects. M1 and M2 are different models in R: M1 = lmer {Death rate ~ [Habitat + Dose]_Fixed effects_ + [(1|Block) + (1|Species richness) + (1|Individual number)]_Random effects_; M2 = lmer (Seeds ~ [Habitat + Dose]_Fixed effects_ + [(1|Block) + (1|Species richness) + (1|Individual number)]_Random effects_}. And “/” means no data.

ANOVA followed by the LSD test were used to compare changes in the relative coverage of *A. artemisiifolia*, native species, and bare ground, and to evaluate community recovery after 2 years of weed control.

All data followed a normal distribution except for the relative cover data of *A. artemisiifolia* after control. For data that did not follow a normal distribution, the Kruskal-Wallis test was used.

## Results

### Herbicide Screening

Aminopyralid was found to be the most effective of the 11 herbicides evaluated (Table 3). At the recommended dose (110 g ai ha^−1^), it resulted in over 90% control of *A. artemisiifolia* plants when applied upon the appearance of male flower buds (BBCH 51); furthermore, it caused a 98% reduction in *A. artemisiifolia* seed yield compared to the control. Glyphosate and glufosinate also reduced seed yield by more than 94%, implying that, *A. artemisiifolia* has not developed glyphosate resistance at this site. Nonetheless, as non-selective herbicides, they were deemed unsuitable for use in habitats with high levels of plant species richness.

### Optimal Period for Herbicide Application

**Figure 2:**
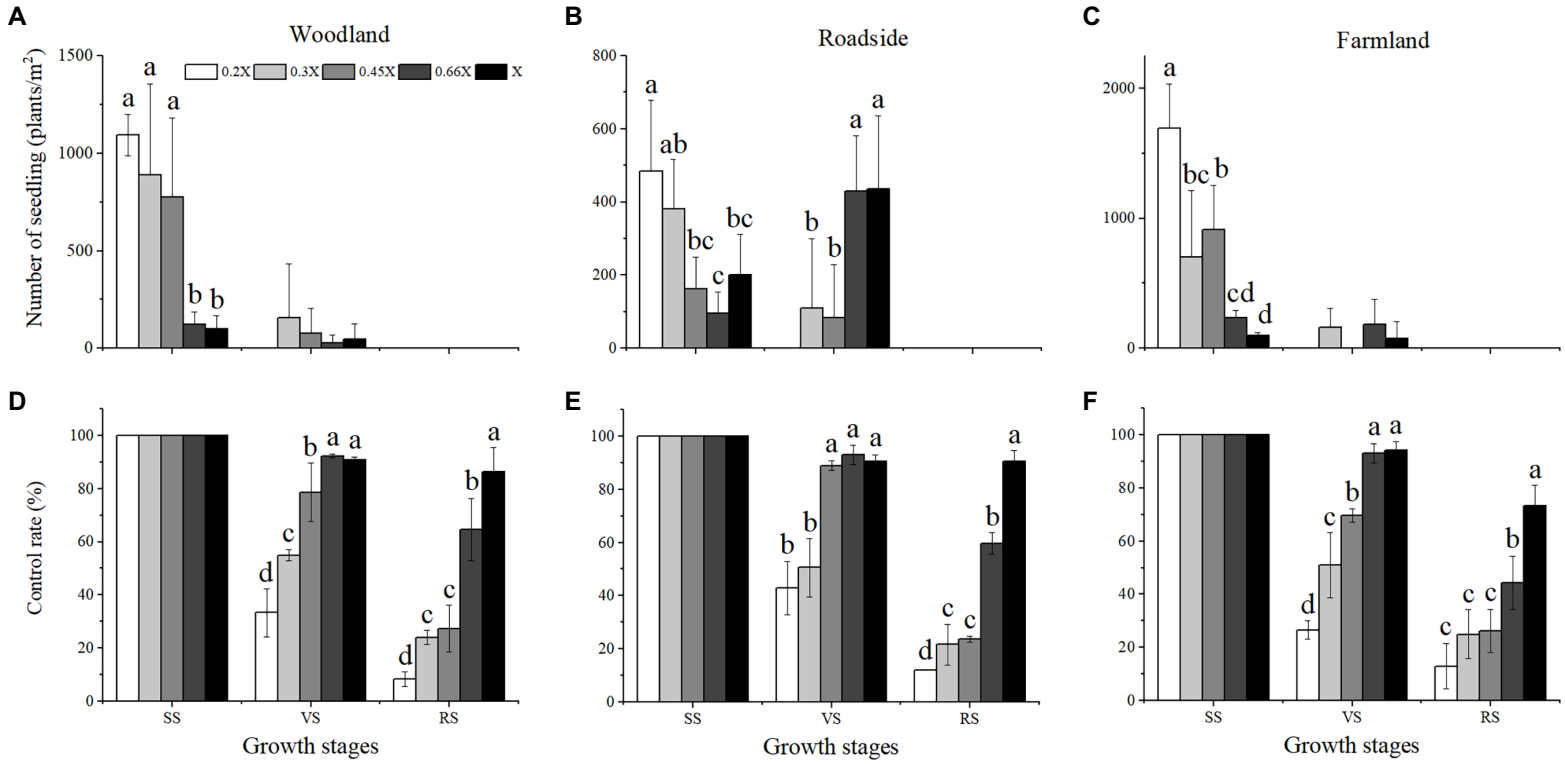
Regeneration **(A–C)** and control rate **(D–F)** of *Ambrosia artemisiifolia* under different treatments. Habitats and growth stages: W, woodland; R, roadside; F, farmland; SS, seedling stage; *VS*, vegetative growth stage; and RS, reproductive growth stage. 0.2X, 0.3X, 0.45X, 0.66X, and X represent different doses of aminopyralid. Intercomparison between different aminopyralid doses within the same treatment time. Different letters indicate significant differences, while treatments without marked letters indicate non-significant differences (*p* < 0.05). Values are means ± SE.

**Figure 3:**
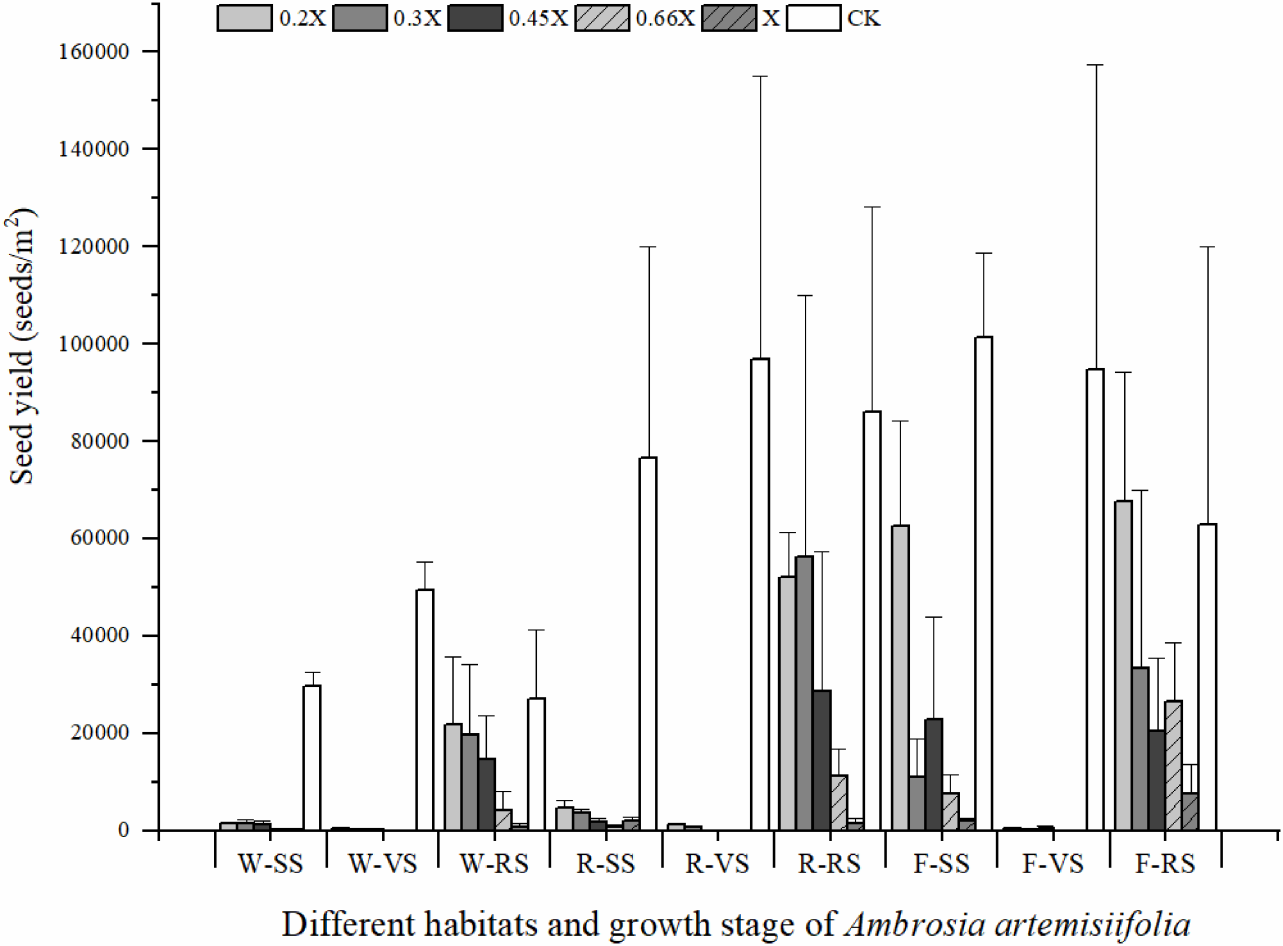
Seed yield of *Ambrosia artemisiifolia* under different treatments. Three treatments in each habitat occurred at different growth stage of *A. artemisiifolia*. Habitats and growth stages: W, woodland; R, roadside; F, farmland; SS, seedling stage; *VS*, vegetative growth stage; RS, reproductive growth stage; and CK, control group. 0.2X, 0.3X, 0.45X, 0.66X, and X represent different doses of aminopyralid. Values are means ± SE.

**Figure 4:**
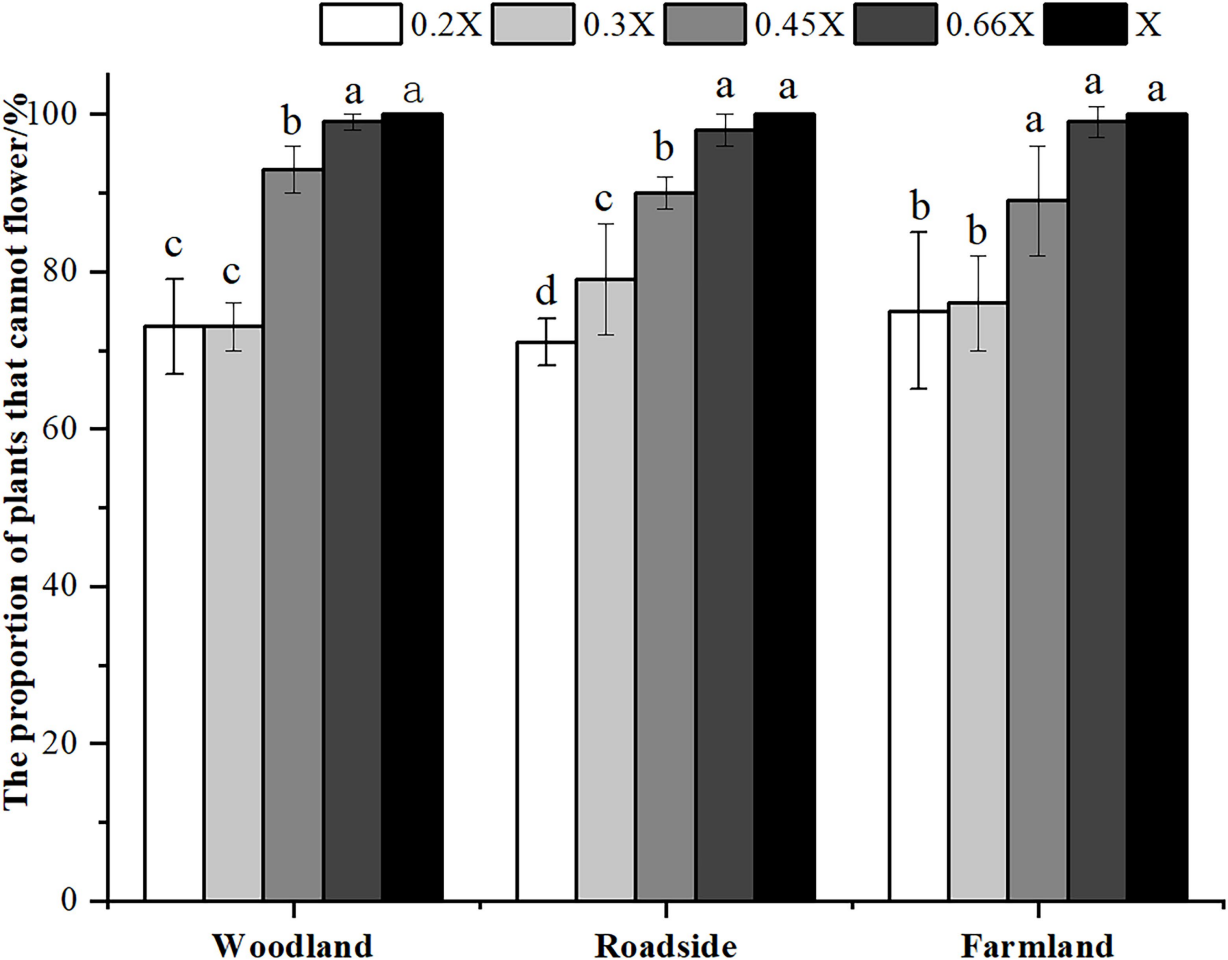
Proportion of plants unable to flower after treatment in the vegetative growth stage (BBCH 32–35) of *Ambrosia artemisiifolia*. Different letters indicate significant differences (*p* < 0.05). 0.2X, 0.3X, 0.45X, 0.66X, and X represent different doses of aminopyralid. Values are means ± SE.

The best time for chemical control of *A. artemisiifolia* was during the vegetative growth stage (BBCH 32–35) in mid-June. Although low-dose (0.2 and 0.3X) applications killed *A. artemisiifolia* plants at the seedling stage (BBCH 14, Figure 2), application during this period also caused the greatest harm to native species, reducing the aboveground biomass of native plants by more than 60% (Supplementary Table 2), while regeneration of *A. artemisiifolia* seedlings was also high (> 800 seedlings/m^2^ on average). Herbicide application during the reproductive growth (BBCH 60–61) period was found to be ineffective at preventing seed production, even at the highest dose tested (Figure 3). Therefore, the optimum growth stage for herbicide application was the vegetative growth stage (BBCH 32–35), when herbicide application was found to inhibit vegetative growth and to reduce the number of plants entering the flowering stage (Figure 4). Thus, at low doses (0.2X and 0.3X), aminopyralid inhibited *A. artemisiifolia* seed production while having minimal effects on native plants species.

### Species Richness and *Ambrosia artemisiifolia* Fitness

**Figure 5:**
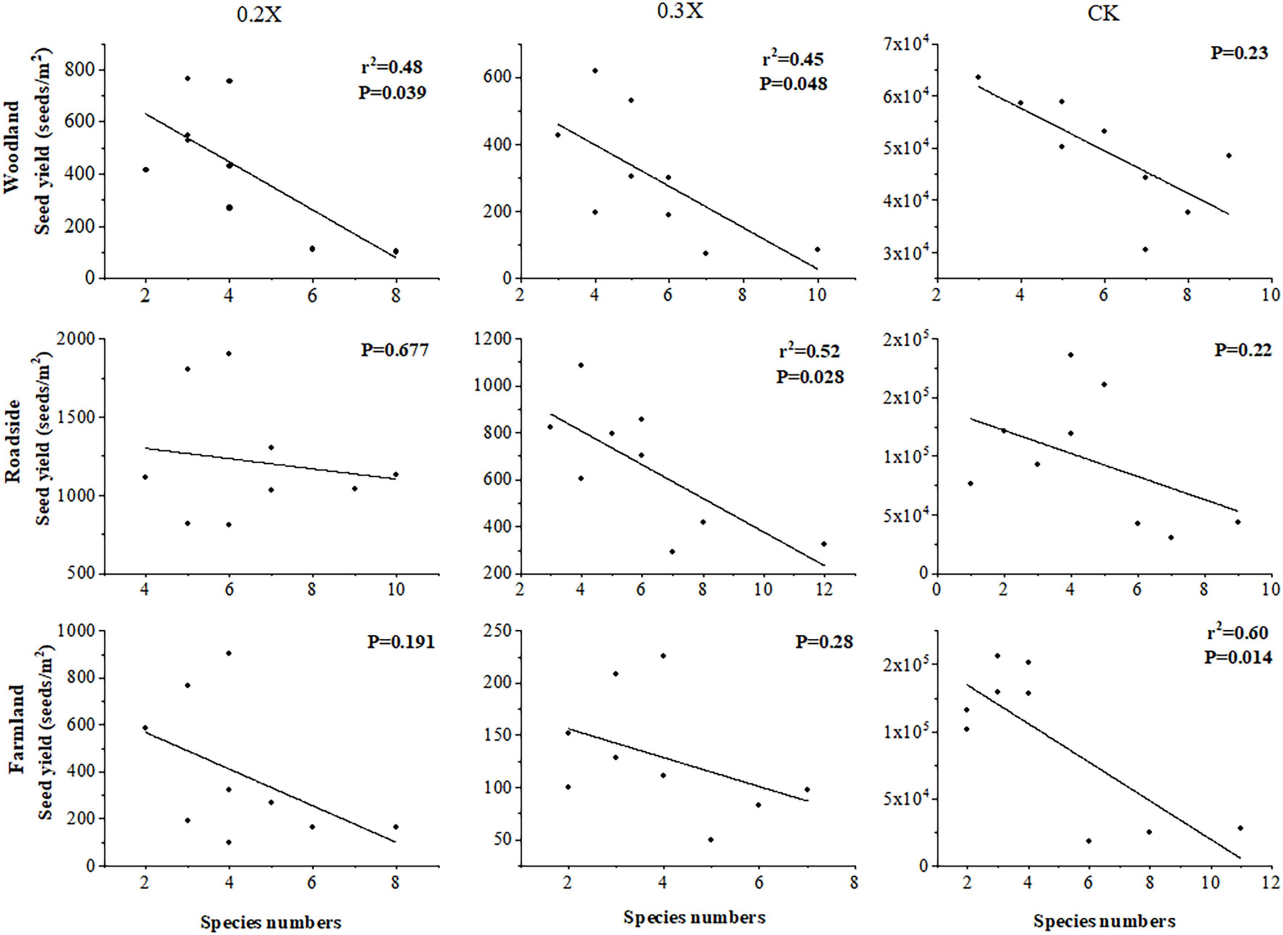
Correlation analysis of native species richness and *Ambrosia artemisiifolia* seed yield. CK, control group, and 0.2X, 0.3X represent different doses of aminopyralid.

With the same dose of aminopyralid, the higher the species richness, the greater the reduction in *A. artemisiifolia* fitness (Table 4). Model M1 indicated that the same dose of aminopyralid did not significantly affect *A. artemisiifolia* control rate, regardless of habitat, whereas species richness of native plants showed a significant effect. The greater the native species richness, the higher the control rate on *A. artemisiifolia*. Results from Model M2 indicated that the seed yield of *A. artemisiifolia* was significantly affected by habitat type and herbicide dose. Further, correlation analysis indicated that with the same dose of aminopyralid, *A. artemisiifolia* seed yield was negatively correlated with species richness (Figure 5).

### Community Recovery

**Figure 6:**
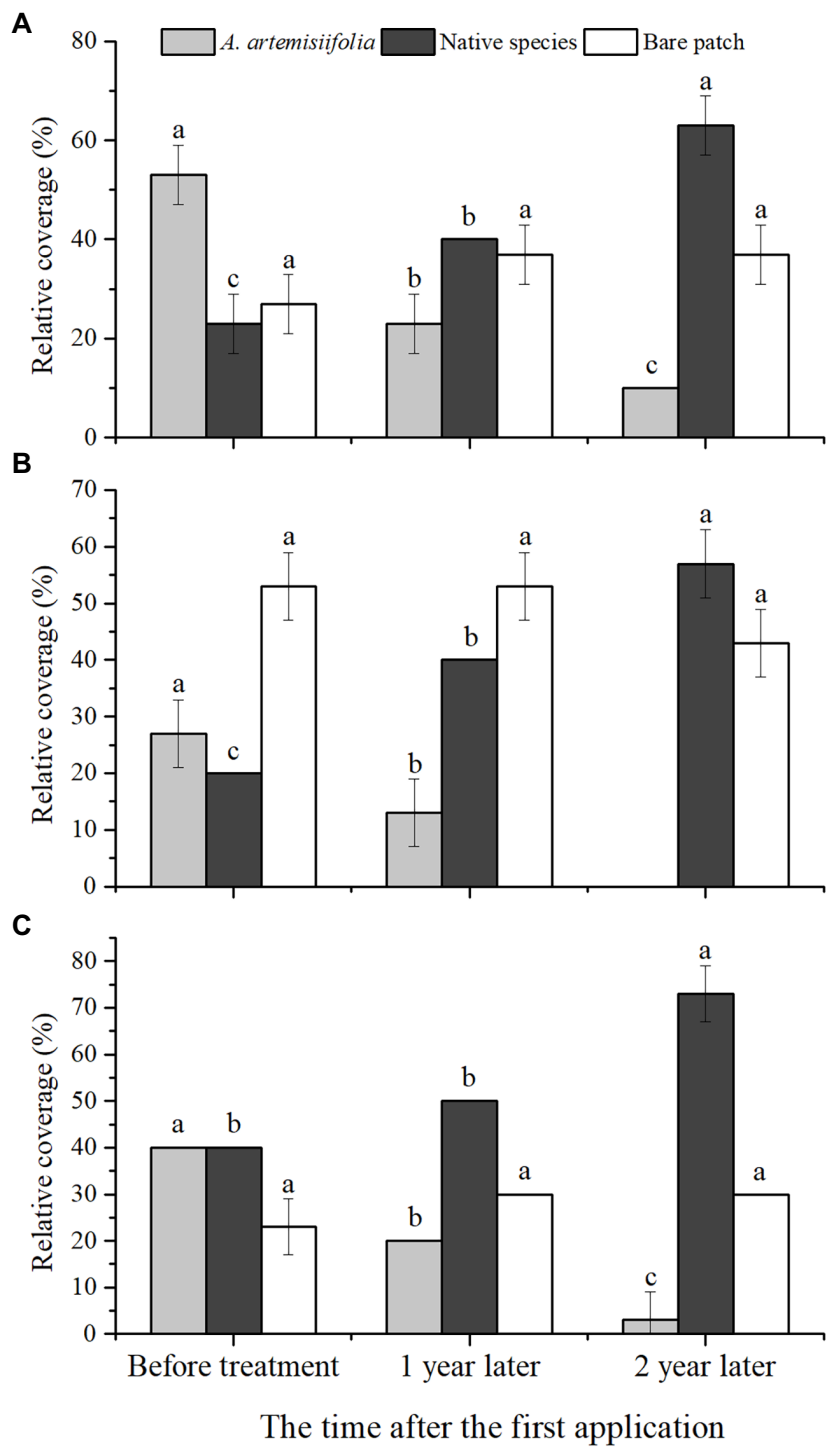
Variation of relative coverage of each component over time in each habitat: woodland **(A)**, roadside **(B),** and farmland **(C)**. Different letters indicate significant differences (*p* < 0.05). Values are means ± SE.

After 2 years of control, the relative coverage of *A. artemisiifolia* significantly decreased and was almost eradicated from the roadside habitat, whereas there was a significant increase in the relative coverage of native species (Figures 1, 6).

## Discussion

We found the effectiveness of herbicide application for *A. artemisiifolia* control to be dependent on plant growth stage. Furthermore, chemical control was found to be most effective during the vegetative growth stage (BBCH 32–35). Therefore, our findings confirm that *A. artemisiifolia* can be controlled in an effective and eco-friendly manner using low herbicide doses. Although this strategy resulted in low mortality rates in *A. artemisiifolia* plants (the lowest was approximately 27%), the treatments inhibited vegetative growth, reproductive growth, pollen production, and seed yield, which was reduced by more than 90%. Without seed replenishment, the soil seed banks can be depleted in a few years, inhibiting invasion by this species and allowing it to be gradually replaced by native species. By evaluating the effectiveness of herbicide treatment in multiple habitats, findings revealed a negative correlation between native species richness and *A. artemisiifolia* fitness, thus providing a basis for highly effective and eco-friendly chemical control of *A. artemisiifolia* in newly invaded areas.

### Seed Yield Reduction Affects Invasiveness of *Ambrosia artemisiifolia*

Invasive annual plants constitute a substantial proportion of the herbaceous invasive plant species found worldwide. In China, annual and biennial herbs together account for 46.1% of invasive plants. More than 60% of invasive plants such as *A. artemisiifolia* can cause severe negative effects on ecosystems (Zhou et al., 2020). The number of seeds and the germination characteristics play key roles in invasion by *A. artemisiifolia*. This species can produce hundreds to tens of thousands of seeds per plant, depending on the habitat and plant size. Seeds also have different dormancy characteristics (Bazzaz, 1974; Baskin and Baskin, 1980), with some seeds of invasive species germinating even after remaining dormant for up to 39 years (Baskin and Baskin, 1980). Propagule pressure has a substantial influence on the success of species invasion (Simberloff, 2009) and is a strong predictor of invasiveness (Colautti et al., 2006). Even in invaded habitats, propagule pressure can sustain invasive populations, and *A. artemisiifolia* can invade a wide variety of habitats after disturbance, thereby accumulating large amounts of biomass.

Our findings suggest that *A. artemisiifolia* seeds can germinate at different times (Figure 2), with germination having a clustering effect, meaning that the propagule pressure of *A. artemisiifolia* may be reflected in the number of seeds that can germinate at the same time. Tens of thousands of *A. artemisiifolia* seedlings can emerge simultaneously per square meter, occupying bare ground, before the numbers rapidly decrease to approximately 1,500–3,000 seedlings/m^2^. Following the death of large numbers of *A. artemisiifolia* plants, additional seeds underground can then germinate rapidly to cover the area. After *A. artemisiifolia* seedlings have been killed by herbicide application, additional seeds in the seed bank germinated quickly, with the new seedlings filling the available space. However, the ability of the seed bank to take advantage of these openings decreased with time. After entering the vegetative growth stage, the number of viable seeds significantly decreased, and after entering the reproductive growth stage, almost no new seedlings appeared. Finally, after 2 years of herbicide application, the relative coverage of *A. artemisiifolia* significantly decreased in the study area (Figure 6).

These results indicate that abundant seed production and high seed germinability constitute a mechanism through which *A. artemisiifolia* can rapidly take advantage of ecological opportunities. Therefore, effective control can be achieved by understanding this germination strategy and depleting the seed bank by inhibiting seed setting through herbicide application at the optimal time.

### Reducing Herbicide Application Rates According to the Developmental Characteristics of *Ambrosia artemisiifolia* Is Important for Sustainable Weed Management

The success of different weed control methods largely depends on the growth stage of the plant at the time of application. Some studies have compared the effectiveness of chemical control at different growth stages of *A. artemisiifolia*, but not much consideration was given to reducing the herbicide dose (Gauvrit and Chauvel, 2010; Bae et al., 2017). For example, Gauvrit and Chauvel (2010) tested herbicides in four stages of the *A. artemisiifolia* life cycle: early vegetative (BBCH 14), bud appearance (BBCH 53–55), pollen production onset (BBCH 61), and mid flowering of female flowers (BBCH 73–77). Using two agents, glufosinate and glyphosate, their study showed that the best time to apply herbicides was at BBCH 53–55. Bae et al. (2017) conducted experiments at stages BBCH 51 and BBCH 61–63 with a total of five agents. Their results showed that the application of tank mixtures containing 2,4-D or dicamba had the potential to limit seed production of glyphosate-resistant *A. artemisiifolia* when applied on or before stage BBCH 51.

While focusing on the chemical control of *A. artemisiifolia*, the weed cannot be considered as a separate element, as it is also under constant pressure by its surrounding environment. To adapt to complex natural environments, plants have evolved sophisticated mechanisms to balance growth and defense responses. The activation of defense mechanisms at the cost of suspending growth is known as the growth–defense tradeoff phenomenon (Coley et al., 1985; Figueroa-Macías et al., 2021). In the absence of pathogens or other environmental stimuli, young tissues must suppress immune or adaptation responses to maximize growth, whereas mature organs can allocate more resources to defense (Wang and Wang, 2014). In the present study, clear growth–defense tradeoffs were observed at different developmental stages in *A. artemisiifolia*. During the vegetative growth stage (BBCH 30–39), *A. artemisiifolia* exhibited rapid growth (Supplementary Table 2). According to this growth–defense tradeoff theory, *A. artemisiifolia* reduces its investment in defense and maximizes growth, resulting in a high level of sensitivity to chemical agents and greater susceptibility to low-dose herbicide treatment. Our findings showed that following treatment, the vertical growth of *A. artemisiifolia* substantially decreased, likely due to the triggering of defense mechanisms. In response to low-dose herbicide treatment, most *A. artemisiifolia* plants survived but to do so, growth and subsequent reproduction were sacrificed. Conversely, herbicide application during the reproductive growth stage did not have this effect, suggesting that the more mature organs had increased defensive capability.

Long-term effective management of *A. artemisiifolia* must rely on considerably reducing seed production to deplete the seed bank. Using this growth–defense tradeoff mechanism for *A. artemisiifolia*, effective control can be rapidly achieved, thus reducing the possible negative effects of the long-term use of low-dose chemicals that might entail the risk of herbicide resistance or plant hormesis (Busi et al., 2013; Jalal et al., 2021). The invasiveness of *A. artemisiifolia* can be reduced primarily by preventing seed production using low herbicide doses and relying on the resilience of native plants. After two years of herbicide treatment, *A. artemisiifolia* was almost entirely eradicated at three sites in the present study (Figure 6). Furthermore, this approach reduces the likelihood of the development of resistance in *A. artemisiifolia* and native plants due to the short-term use of low-dose herbicides. Thus, in the third year (2021), when no herbicide was applied, native plants effectively occupied the habitat (Figures 1, 6), demonstrating that this method is highly efficient and eco-friendly.

Reduction of *A. artemisiifolia* seed production, or even completely inhibiting it, means that the number of offspring that can develop resistance is reduced, and when combined with government increasing phytosanitary policies, the risk of spreading resistant offspring is greatly reduced. In the worst-case scenario, even if *A. artemisiifolia* becomes resistant to the herbicide, we can still increase the dose of the herbicide, as we would be using a low dose. This means that the duration of an effective agent can be extended, thus allowing the necessary time for the discovery or development of new agents.

### High Species Richness Can Enhance the Efficacy of Herbicides

In addition to directly reducing the fitness of *A. artemisiifolia*, herbicide application also mediated interspecific competition with native plants, which indirectly reduced the fitness of *A. artemisiifolia* (Table 4; Figure 5). According to the biotic resistance hypothesis (Elton, 1958; Levine and D'Antonio, 1999; Jeschke et al., 2012; Henriksson et al., 2016), plant communities with high species diversity are more resistant to invasion by invasive species than communities with low species diversity, with species diversity being negatively correlated with invasion. This hypothesis has been supported experimentally and theoretically by some studies (Dukes, 2002; Kennedy et al., 2002; Beaury et al., 2020). Mwangi et al. (2007) tested the hypothesis by transplanting species into experimental grassland communities, and showed that invasion resistance is related to the degree of niche overlap between resident species and invaders. This niche overlap can be high due to generally low amounts of empty niche space in species-rich resident communities. However, there are cases that do not support this hypothesis (Levine et al., 2004; Akin-Fajiye et al., 2021). Collins et al. (2007) found that species diversity does not appear to be an important factor for *Imperata cylindrica* invasion in the southern United States, and reasons for why no relationship was observed may be simply due to the tremendous competitive ability of *I. cylindrica*. At present, this is controversial.

Our study better fit here. On a small scale, high species richness reduced *A. artemisiifolia* fitness. The richness of native species was negatively correlated with *A. artemisiifolia* seed yield, regardless of herbicide treatment. It is likely that plant species in more diverse communities occupy a wider range of ecological niches and are more apt to compete with *A. artemisiifolia* (Byun et al., 2013, 2020). Diversified interspecific competition largely reduces the fitness of *A. artemisiifolia*, thereby providing a solid basis for effective weed control using herbicides at low doses prior to *A. artemisiifolia* outbreaks.

## Data Availability Statement

The raw data supporting the conclusions of this article will be made available by the authors, without undue reservation.

## Author Contributions

HW and TL conceived the ideas and designed methodology and led the writing of the manuscript. HW, WZ, XL, PS, MS, and JW collected the data. HW analyzed the data. All authors contributed critically to the drafts and gave final approval for publication.

## Funding

This work was supported by the National Natural Science Foundation of China (31770461).

## Conflict of Interest

The authors declare that the research was conducted in the absence of any commercial or financial relationships that could be construed as a potential conflict of interest.

## Publisher’s Note

All claims expressed in this article are solely those of the authors and do not necessarily represent those of their affiliated organizations, or those of the publisher, the editors and the reviewers. Any product that may be evaluated in this article, or claim that may be made by its manufacturer, is not guaranteed or endorsed by the publisher.
