# Supplementary material for: Reduced Invasiveness of Common Ragweed (Ambrosia artemisiifolia) Using Low-Dose Herbicide Treatments for High-Efficiency and Eco-Friendly Control
Source: Front Plant Sci. 2022 May 12;13:861806. doi: 10.3389/fpls.2022.861806 (PMC9133841; doi:10.3389/fpls.2022.861806)
Supplement: Supplementary file 1 [file Table_1.DOCX]

**Supplementary Table 1.** Species in each habitats.

| Habitats | Families | Species | Life form |
| --- | --- | --- | --- |
| Woodland | Compositae | *Xanthium sibiricum* | annual |
|  |  | *Artemisia carvifolia* | annual |
|  |  | *Arctium lappa* | biennial |
|  |  | *Taraxacum mongolicum* | perennial |
|  |  | *Lactuca sativa* | annual or biennial |
|  |  | *Conyza canadensis* | annual |
|  | Labiatae | *Phlomis umbrosa* | perennial |
|  |  | *Glechoma longituba* | perennial |
|  |  | *Mentha haplocalyx* | perennial |
|  | Gramineae | *Bromus japonicus* | annual |
|  |  | *Festuca ovina* | perennial |
|  | Leguminosae | *Trifolium repens* | perennial |
|  |  | *Medicago sativa* | perennial |
|  | Gramineae | *Lolium perenne* | perennial |
|  | Plantaginaceae | *Plantago asiatica* | perennial |
|  | Rosaceae | *Potentilla chinensis* | perennial |
|  | Brassicaceae | *Brassica juncea* | annual |
|  | Compositae | *Onopordum acanthium* | biennial |
|  | Geraniaceae | *Geranium wilfordii* | perennial |
|  | Moraceae | *Cannabis sativa* | annual |
| Roadside | Compositae | *Xanthium sibiricum* | annual |
|  |  | *Onopordum acanthium* | biennial |
|  |  | *Erigeron acer* | biennial |
|  |  | *Arctium lappa* | biennial |
|  |  | *Taraxacum mongolicum* | perennial |
|  |  | *Ambrosia trifida* | annual |
|  |  | *Conyza canadensis* | annual |
|  |  | *Crassocephalum crepidioides* | annual or biennial |
|  | Leguminosae | *Medicago sativa* | perennial |
|  |  | *Trifolium pratense* | perennial |
|  |  | *Tephrosia candida* | perennial |
|  | Gramineae | *Echinochloa crusgali* | annual |
|  |  | *Phragmites australis* | perennial |
|  |  | *Bromus japonicus* | annual |
|  | Compositae | *Sonchus oleraceus* | annual or biennial |
|  | Plantaginaceae | *Plantago asiatica* | perennial |
|  | Brassicaceae | *Rorippa indica* | annual or biennial |
|  | Equisetaceae | *Equisetum ramosissimum* | perennial |
|  | Geraniaceae | *Geranium wilfordii* | perennial |
|  | Moraceae | *Cannabis sativa* | annual |
|  | Rosaceae | *Geum aleppicum* | perennial |
|  | Umbelliferae | *Daucus carota* | biennial |
| Farmland | Compositae | *Arctium lappa* | biennial |
|  |  | *Taraxacum mongolicum* | perennial |
|  |  | *Lagedium sibiricum* | perennial |
|  |  | *Conyza canadensis* | annual |
|  | Gramineae | *Echinochloa crusgali* | annual |
|  |  | *Lolium perenne* | perennial |
|  |  | *Eleusine indica* | annual |
|  |  | *Bromus japonicus* | annual |
|  | Leguminosae | *Medicago sativa* | perennial |
|  |  | *Trifolium pratense* | perennial |
|  | Plantaginaceae | *Plantago asiatica* | perennial |
|  | Rosaceae | *Potentilla chinensis* | perennial |
|  | Boraginaceae | *Myosotis silvatica* | perennial |
|  | Brassicaceae | *Arabidopsis thaliana* | biennial |
|  | Chenopodiaceae | *Chenopodium album* | annual |
|  | Labiatae | *Prunella asiatica* | annual |
|  | Moraceae | *Cannabis sativa* | annual |
|  | Polygonaceae | *Rumex acetosa* | perennial |
