# Supplementary material for: Reduced Invasiveness of Common Ragweed (Ambrosia artemisiifolia) Using Low-Dose Herbicide Treatments for High-Efficiency and Eco-Friendly Control
Source: Front Plant Sci. 2022 May 12;13:861806. doi: 10.3389/fpls.2022.861806 (PMC9133841; doi:10.3389/fpls.2022.861806)
Supplement: Supplementary file 2 [file Table_2.DOCX]

**Supplementary Table 2.** Effects of herbicide on growth of *Ambrosia artemisiifolia* and indigenous species.

| Habitats | Growth stages | Concentration of herbicide | Plant Height of *A. artemisiifolia* | Reduced above-ground biomass of *A. artemisiifolia* | Reduced above-ground biomass of  indigenous plants |
| --- | --- | --- | --- | --- | --- |
| woodland | seedling stage | 0.2X | 5.4±2.9 c | 34.79%±38.38% c | 57.51%±43.25% b |
|  |  | 0.3X | 5.4±2.9 c | 58.97%±16.75% b | 70.96%±17.64% ab |
|  |  | 0.45X | 7.2±1.9 bc | 37.71%±40.31% c | 84.65%±8.79% a |
|  |  | 0.66X | 9.1±2.7 b | 88.63%±6.19% a | 84.09%±9.70% a |
|  |  | X | 5.2±1.7 c | 94.81%±3.52% a | 89.77%±5.98% a |
|  |  | CK | 12.7±3.2 a | / | / |
|  | vegetative growth stage | 0.2X | 16±4.9 c | 13.52%±43.96% d | 12.30%±22.01% c |
|  |  | 0.3X | 20.8±6.5 b | 55.97%±32.55% c | 6.97%±33.60% c |
|  |  | 0.45X | 20.9±2.2 b | 84.10%±17.85% b | 62.64%±8.23% a |
|  |  | 0.66X | 19.1±7.7 b | 100.00%±0.00% a | 42.32%±27.54% b |
|  |  | X | 17.7±5.8 c | 100.00%±0.00% a | 57.39%±27.56% a |
|  |  | CK | 35.7±1.5 a | / | / |
|  | reproductive growth stage | 0.2X | 28.6±6.7 a | -6.92%±11.14% e | 50.14%±5.26% b |
|  |  | 0.3X | 33.3±8.1 a | 23.47%±40.05% d | 45.98%±8.94% c |
|  |  | 0.45X | 30.1±3.6 a | 55.05%±31.38% c | 64.70%±8.82% a |
|  |  | 0.66X | 30.8±3.9 a | 79.33%±7.12% b | 46.90%±26.38% c |
|  |  | X | 29.1±9.3 a | 96.99%±3.81% a | 65.00%±19.49% a |
|  |  | CK | 31.7±6.1 a | / | / |
| roadside | seedling stage | 0.2X | 8.2±1.4 bc | -46.31%±20.09% e | 65.72%±12.41% c |
|  |  | 0.3X | 8.3±4.1 bc | -25.87%±105.49% d | 79.82%±5.66% ab |
|  |  | 0.45X | 9.5±1.4 b | 56.39%±11.96% c | 70.16%±15.87% b |
|  |  | 0.66X | 7.1±0.5 c | 78.34%±14.67% a | 83.50%±5.19% a |
|  |  | X | 6.1±2 d | 68.50%±9.93% b | 88.50%±9.64% a |
|  |  | CK | 15.2±2.5 a | / | / |
|  | vegetative growth stage | 0.2X | 37.6±3.2 c | 44.93%±30.26% b | -0.01%±11.94% d |
|  |  | 0.3X | 35.5±8.3c | 44.33%±28.04% b | 10.61%±49.26% c |
|  |  | 0.45X | 43.3±13.7 b | 97.68%±4.02% a | -21.10%±72.20% e |
|  |  | 0.66X | 41.9±6.5 b | 100.00%±0.00% a | 28.51%±27.41% b |
|  |  | X | 31.6±9 c | 100.00%±0.00% a | 51.56%±24.58% a |
|  |  | CK | 57.1±6.4 a | / | / |
|  | reproductive growth stage | 0.2X | 49.7±6.6 b | -20.03%±58.57% d | -38.29%±20.43% d |
|  |  | 0.3X | 51.6±2.2 ab | 19.98%±46.43% c | -9.31%±58.44% c |
|  |  | 0.45X | 57.5±16.4 a | 67.71%±18.60% b | -50.05%±96.18% e |
|  |  | 0.66X | 49.8±7.1 b | 54.18%±30.09% b | 22.39%±44.78% b |
|  |  | X | 44.8±17.6 c | 93.07%±8.91% a | 51.85%±24.12% a |
|  |  | CK | 56.3±9.3 a | / | / |
| farmland | seedling stage | 0.2X | 12.3±1.2 b | -63.20%±79.55% d | 62.73%±6.61% b |
|  |  | 0.3X | 13±2.7 b | 33.99%±42.03% c | 67.21%±21.92% b |
|  |  | 0.45X | 12±1.2 b | 20.96%±32.65% c | 80.68%±15.36% a |
|  |  | 0.66X | 12.8±1.4 b | 73.23%±15.55% b | 89.44%±4.42% a |
|  |  | X | 11.6±5.1 b | 92.94%±0.09% a | 87.59%±11.64% a |
|  |  | CK | 30.2±4.9 a | / | / |
|  | vegetative growth stage | 0.2X | 85.1±3.1 b | -62.00%±91.03% d | -8.25%±29.13% d |
|  |  | 0.3X | 59.7±6.2 d | 5.63%±59.44% c | 20.07%±6.34% b |
|  |  | 0.45X | 62.6±10.5 cd | 56.98%±20.85% b | 9.35%±64.47% c |
|  |  | 0.66X | 67.6±9.7 c | 100.00%±0.00% a | 57.88%±13.07% a |
|  |  | X | 75.8±12.1 bc | 100.00%±0.00% a | 52.31%±33.75% a |
|  |  | CK | 102.6±14 a | / | / |
|  | reproductive growth stage | 0.2X | 106.8±9.6 a | -65.99%±143.10% e | -23.05%±76.57% c |
|  |  | 0.3X | 80.5±2 c | 3.02%±70.40% c | 36.40%±17.40% b |
|  |  | 0.45X | 82.1±11.7 c | 26.39%±30.88% b | -18.04%±114.11% c |
|  |  | 0.66X | 94±21.9 b | -27.47%±141.86% d | 67.27%±13.34% a |
|  |  | X | 106.6±4.2 a | 62.25%±25.65% a | 61.01%±17.63% a |
|  |  | CK | 100.5±23.2 a | / | / |

Values in table are Means±SE. Different letters indicate significant differences (p < 0.05) using a least significant difference test. CK means control group. And 0.2X, 0.3X, 0.45X, 0.66X and X represent different doses of aminopyralid. And “/” means no data.
